# Effect of flow and cannula size on generated pressure during nasal high flow

**DOI:** 10.1186/s13054-020-02980-w

**Published:** 2020-05-24

**Authors:** Maximilian Pinkham, Stanislav Tatkov

**Affiliations:** Fisher & Paykel Healthcare Ltd, PO Box 14 348, Auckland, 1741 New Zealand

**Keywords:** Nasal high flow, Nasal cannula, Positive airway pressure, Acute respiratory failure, COVID-19

## Introduction

Nasal high flow (NHF) with supplemental oxygen has been proposed to treat COVID-19 patients with acute respiratory failure (ARF) [1]. NHF gained its popularity due to the high oxygenation efficiency and success in treating patients with hypoxemic ARF [2]. Apart from the delivery of oxygen, NHF reduces the rebreathing from anatomical dead space and generates positive airway pressure; however, the delivered pressure is difficult to quantify due to the unsealed cannula interface. The purpose of this research is to provide clinicians with the indicative data of the generated pressure using different flow settings and cannula sizes.

## Methods

A chamber with two adjacent orifices to fit the cannula served as a model of the nasal cavity with nares, which has been described in detail previously [3]. Briefly, two “nare” sizes of 9-mm and 10-mm diameter were included to replicate similar pressures observed previously in patients [4]. NHF was delivered via a smaller-sized cannula, O.D. 6.1 mm/I.D. 5.1 mm, and a larger-sized cannula, O.D. 7.2 mm/I.D. 6.0 mm (Optiflow™ OPT944 “Medium” and OPT946 “Large”, Fisher & Paykel Healthcare, NZ). Measurements were made during the static condition to replicate pressure at the end of expiration in a mouth-closed position.

## Results

**Fig. 1:**
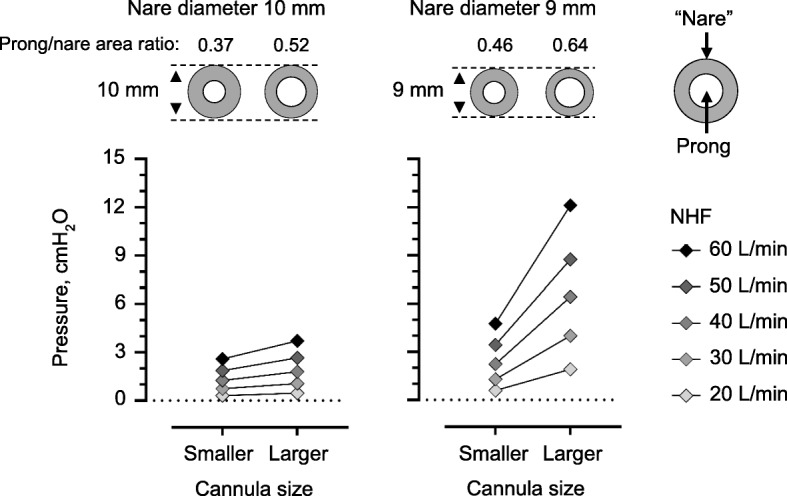
The graph shows the positive airway pressure, cmH2O, that is generated by nasal high flow (NHF) using a smaller cannula (O.D. 6.1 mm) and larger cannula (O.D. 7.2 mm) in “nares” with two different diameters: 10 mm (left panel) and 9 mm (right panel) in the bench top model. Pressure generated by NHF can be increased by higher flow and by occluding a larger area of the nare, which can be achieved by increasing the cannula size

As shown in Fig. 1, NHF generates greater pressure when delivered using a larger cannula and with higher flow rate. For example, NHF of 60 L/min generated pressure of 12.1 cmH_2_O using the larger cannula compared to 4.8 cmH2O using the smaller cannula, in the 9-mm diameter “nare”. The same cannula size will generate greater pressure when used in a smaller nare. Therefore, using higher flow rates as well as increasing the prong/nare area ratio will generate greater positive airway pressure.

## Discussion

The data show that in order to deliver higher pressure during NHF, then the flow rate and/or cannula size need to be increased. The results are taken from a bench experiment with inelastic orifices, and patients rarely have a closed system; however, the bench data demonstrate that a small reduction of the leak around the cannula, by occluding a larger area of the nare, may lead to a substantial increase of delivered pressure, particularly in the upper range of NHF rates.

The relationship between the occlusion and flow rate in generating positive airway pressure is non-linear; as the occlusion is increased, then pressure will increase significantly due to the increased resistance to flow leaving the nare [3]. Using a very small internal diameter of cannula may also affect relationship between flow and pressure through the jetting effect; however, this was outside the scope of the study [5].

The increase of pressure may reduce comfort and encourage a patient to open their mouth, which may lead to an increase of leak and reduction of pressure [4]; there is no data on the clinical effects of opening the mouth in patients with ARF. Also, the opening of the mouth may enhance the dead space clearance [6]. Larger-sized cannula may potentially reduce the clearance due to the lower leak if the mouth is not opened. However, at the highest flow rate, the dead space clearance is likely to be maximized regardless of the cannula size. Therefore, at 60 L/min, which is 1000 mL/s, the nasal cavity will be filled with fresh gas within a fraction of a second leading to rapid dilution and purging of the expired air.

The experimental data demonstrates the rise in pressure with the increase of the cannula size and flow rate. The findings can be of practical value in the management of patients with severe ARF who need higher levels of positive airway pressure.

## Data Availability

All data generated or analyzed during this study are included in this published article.
